# How artificial intelligence can provide information about subdural hematoma: Assessment of readability, reliability, and quality of ChatGPT, BARD, and perplexity responses

**DOI:** 10.1097/MD.0000000000038009

**Published:** 2024-05-03

**Authors:** Şanser Gül, İsmail Erdemir, Volkan Hanci, Evren Aydoğmuş, Yavuz Selim Erkoç

**Affiliations:** a Department of Neurosurgery, Ankara Ataturk Sanatory Education and Research Hospital, Ankara, Turkey; b Department of Anesthesiology and Critical Care, Faculty of Medicine, Dokuz Eylül University, Izmir, Turkey; c Department of Anesthesiology and Reanimation, Ankara Sincan Education and Research Hospital, Ankara, Turkey; d Department of Neurosurgery, Istanbul Kartal Dr Lütfi Kırdar City Hospital, Istanbul, Turkey.

**Keywords:** artificial intelligence, Bard, ChatGPT, online medical information, perplexity, readability, subdural hematoma

## Abstract

Subdural hematoma is defined as blood collection in the subdural space between the dura mater and arachnoid. Subdural hematoma is a condition that neurosurgeons frequently encounter and has acute, subacute and chronic forms. The incidence in adults is reported to be 1.72–20.60/100.000 people annually. Our study aimed to evaluate the quality, reliability and readability of the answers to questions asked to ChatGPT, Bard, and perplexity about “Subdural Hematoma.” In this observational and cross-sectional study, we asked ChatGPT, Bard, and perplexity to provide the 100 most frequently asked questions about “Subdural Hematoma” separately. Responses from both chatbots were analyzed separately for readability, quality, reliability and adequacy. When the median readability scores of ChatGPT, Bard, and perplexity answers were compared with the sixth-grade reading level, a statistically significant difference was observed in all formulas (*P* < .001). All 3 chatbot responses were found to be difficult to read. Bard responses were more readable than ChatGPT’s (*P* < .001) and perplexity’s (*P* < .001) responses for all scores evaluated. Although there were differences between the results of the evaluated calculators, perplexity’s answers were determined to be more readable than ChatGPT’s answers (*P* < .05). Bard answers were determined to have the best GQS scores (*P* < .001). Perplexity responses had the best Journal of American Medical Association and modified DISCERN scores (*P* < .001). ChatGPT, Bard, and perplexity’s current capabilities are inadequate in terms of quality and readability of “Subdural Hematoma” related text content. The readability standard for patient education materials as determined by the American Medical Association, National Institutes of Health, and the United States Department of Health and Human Services is at or below grade 6. The readability levels of the responses of artificial intelligence applications such as ChatGPT, Bard, and perplexity are significantly higher than the recommended 6th grade level.

## 1. Introduction

Subdural hematoma (SDH) is defined as blood collection in the subdural space between the dura mater and arachnoid.^[1]^ Subdural hematoma is a condition that neurosurgeons frequently encounter and has acute, subacute and chronic forms.^[2]^ Acute subdural hematoma is reported as the most common intracranial traumatic lesion requiring surgery, especially in high-income countries.^[3]^ Chronic subdural hematoma is defined as blood-containing serous fluid collection in the subdural space between the dura mater and arachnoid that develops within 3 or more weeks from the first spontaneous or post-traumatic bleeding event.^[4–6]^ The incidence in adults is reported to be 1.72–20.60/100.000 people annually. Studies have reported an annual incidence of up to 129.5/100.000 in people over the age of 80. Compared to past studies, its incidence is increasing over the years.^[4–8]^ It has been reported that increase in antithrombotic use, aging of the population, alcoholism, neurological and systemic diseases due to brain atrophy, hemodialysis, low intracranial pressure and dural rupture associated with an increased risk of SDH.^[5,8]^ The clinical presentation of SDH is highly variable. Patients typically present with confusion, headache, and loss of consciousness. Symptoms can vary widely between patients. Some patients are asymptomatic. Some present with symptoms such as headache, gait instability, cognitive impairment, or focal neurological deficits. Others have a severe clinical picture with stupor and even coma. Because symptoms are nonspecific, diagnosis is usually confirmed by noncontrast computed tomography or, more rarely, magnetic resonance imaging. SDH appears as a crescent-shaped mass with a hypodense to isodense consistency on cranial computed tomography.^[5–10]^ Treatment for SDH can be conservative or surgical, depending on symptoms and clot size. Complications and relapses are reported to be common. It is emphasized that there is no consensus on the best surgical technique to use. The recurrence rate of SDH after surgery varies between 5% and 30%.^[5,7,9,10]^

The technology that aims to minimize human intervention and in which computer technology is at the forefront is called Artificial Intelligence (AI). Nowadays, the interest and number of studies on artificial intelligence are increasing. Artificial intelligence has begun to be used in many areas of science, technology and health. Artificial intelligence techniques are divided into different branches. Knowledge-based expert system approach, artificial neural networks approach, fuzzy logic approach, nontraditional optimization techniques, object-oriented programming, geographic information systems, decision support systems, soft computing are among these.^[11–13]^

With the development of artificial intelligence, artificial intelligence techniques such as Bayesian networks, fuzzy expert systems, artificial neural networks and hybrid intelligent systems have begun to be used in different clinical settings in health services. Compared to other sectors, healthcare applications account for the largest portion of investments in artificial intelligence research.^[11,13–15]^ Artificial intelligence applied in the field of medicine is divided into 2 groups as physical and virtual. The physical part includes examples such as robots that assist during surgeries, care for the elderly, or smart prosthetics for people with disabilities. The virtual part consists of applications such as neural network-based guidance and electronic health record systems.^[15]^ Among the applications of artificial intelligence in the field of medical sciences, there are applications such as choosing a doctor according to patient symptoms, patient diagnosis, patient prognosis and drug discovery.^[16]^

As another artificial intelligence application, there are widely used artificial intelligence chat programs, namely Chat GPT, Bard, and perplexity, with which the society interacts the most today. ChatGPT is a chatbot developed by OpenAI on November 30, 2022. This chatbot has been developed as a program that can understand the language in which it is written, while answering the questions asked to it. The difference from the Google search engine is that it does not specify a bibliography and the answer can be reached more quickly.^[17]^ GPT is defined as Generative Pre-trained Transformer and uses the GPT-3.5 language model. The premium version, ChatGPT Plus, uses the newly developed GPT-4 model, but is paid. Through Language Models for Dialogue Applications, Google introduced and launched Bard, an experimental conversational AI, in February 2023. These programs represent a family of Transformer-based neural language models, distinguished by their impressive scale with up to 137 billion parameters.^[18]^

Educational materials for patients help patients understand their acute or chronic health conditions. For this reason, more and more patients and their relatives are benefiting from internet-based patient education materials (PEM) for their diseases and health information.^[19,20]^

Chatbots provide easy access to information. They offer personal interaction. It acts as an interactive platform. For these reasons, it is gaining more and more popularity in healthcare services.^[21]^ These technologies such as Google’s Bard and Open AI’s ChatGPT are emphasized as an important tool to increase the safety and quality of healthcare services.^[22]^ However, some concerns may arise with this technology, such as accuracy and readability.^[21,22]^

Readability is numerical values determined by systemic formulas that reflect the reading level required to understand written materials. Many formulas and calculators have been created to determine the readability level. The readability standard for patient education materials as determined by the American Medical Association, National Institutes of Health (NIH), and the United States Department of Health and Human Services is at or below grade 6.^[23–25]^

Our hypothesis in this study is that the readability values of answers which produced by the AI chatbot ChatGPT, Bard, and perplexity according patients’ questions related to Subdural Hematoma are not within the recommended limits. To test this hypothesis, we aimed to evaluate the readability of answers to questions asked ChatGPT, Bard, and perplexity about subdural hematoma using scores.

## 2. Materials methods

This original research study was planned as an observational, cross-sectional study. For this study, 2 independent authors (İ.E. and V.H.) investigated the term “Subdural Hematoma” on ChatGPT, Bard, and perplexity on January 3, 2024. Two independent authors (İ.E. and V.H.) asked ChatGPT, Bard, and perplexity separately “what are most frequently asked top 100 questions about Subdural Hematoma.” The questions asked to chatbots were evaluated and similar or duplicate questions were eliminated. After identifying the 100 most frequently asked questions, each question was asked to ChatGPT, Bard, and perplexity.^[26,27]^ One hundred questions were asked to each of the 3 artificial intelligences (ChatGPT, Bard, and perplexity) separately and the 100 answers obtained for each of the 3 artificial intelligences were recorded. All questions and answers were recorded on the internet (access address: https://archive.org/download/subdural-hematom-questions-and-answers). Responses from chatbots were analyzed separately.

### 2.1. Readability assessment

The texts was copied and saved in Microsoft Office Word 2007 (Microsoft Corporation, Redmond, WA). Two calculators were used to evaluate the readability of the websites. Calculator 1: https://readabilityformulas.com/free-readability-formula-tests.php, and Calculator 2: https://www.online-utility.org/english/readability_test_and_improve.jsp. Texts were evaluated using both calculators.^[25]^ By adding the readability formula results obtained from both calculators separately and dividing them by 2, the readability results for the formulas were determined.^[25]^ The Flesch Reading Ease Score (FRES), Flesch–Kincaid grade level (FKGL), Simple Measure of Gobbledygook (SMOG), gunning FOG (GFOG), Coleman–Liau score (CL), automated readability index (ARI), and Linsear write were used as readability formulas.^[23–25,28–30]^ Calculation explanations of the formulas are shown in Table 1. Different readability scores of 100 questions determined for all 3 artificial intelligences were determined and median (minimum–maximum) values were obtained. The readability scores were compared and analyzed with the sixth-grade level of readability recommended by the American Medical Association and NIH. The accepted readability level in the FRES formula was 80.0, while it was 6 for the other 6 formulas.^[23–25,28–30]^

**Table 1:** Readability index formulas.

Readability index	Formula
Flesch Reading Ease Score (FRES)	**I =** (206.835 - (1.015 **X** (W**/** S)) - (84.6 **X** (B/ W)))
Gunning FOG (GFOG)	**G = **0.4 **X** (W**/** S**+**((C**/** W) **X** 100))
Flesch–Kincaid grade level (FKGL)	**G =** (11.8 **X** (B**/** W)) **+** (0.39 **X** (W/ S)) **−**15.59
Simple Measure of Gobbledygook (SMOG)	**G =** 1.043 **X √ C +** 3.1291
Coleman–Liau (CL) score	**G =** (0.0588 **X** K) - (0.296 **X** L)–15.8
Automated readability index (ARI)	**ARI =** (4.71 **X** (J**/** W)) **+** (0.5 **X** (W**/** S)) **−**21.43
Linsear Write (LW)	**LW =** (ASL **+ **(2 **X** HDW))**/** S
Forcast readability formula (FRF)	G = 20–((NWS**/** 150)**/** (W **X** 10))

ASL = average sentences length (number of words divided by the number of sentences), B = number of syllables, C = complex words (≥3 syllables), G = grade level, HDW = number of “hard words” (words with more than 2 syllables), I = Flesch Index Score, J = number of characters, K = average number of characters per 100 words, L = average number of sentence per 100 words, NWS = number of single syllable words, S = number of sentences, W = number of words.

### 2.2. Reliability assessment

Journal of American Medical Association (JAMA) criteria were used to determine the reliability. JAMA criteria; providing authors and contributors, their affiliations and relevant identification information; listing references and sources for all content; It consists of 4 items: full disclosure of conflicts of interest, financing, sponsorship, advertising, support and text ownership, and indicating the dates the content was published and updated, and the articles receive 0 or 1 point for each item.^[23–25,28–30]^

### 2.3. Quality assessment

Modified DISCERN and Global Quality Scale (GQS) were used to evaluate the quality. GQS is a scoring system which is scored between 1 to 5 and consisting of the following items: “Excellent quality and flow, very helpful for patients,” “Good quality and flow, helpful for patients,” “Fair quality, suboptimal flow, somewhat helpful for patients,” “Very limited use for patients, poor quality and poor flow overall,” “Poor quality, poor flow, not at all useful for patients.” Modified DISCERN is a scoring system that includes 5 questions and is calculated by giving 0 or 1 point for each question depending on whether these questions are met. Since there were 2 independent authors in this study, modified DISCERN and GQS scores were evaluated by calculating the average of the scores given by the authors.^[23–25,28–30]^

### 2.4. Ethical considerations

The study includes a methodology that does not require the use of human participants, human or animal data. The study includes anonymized data collected on the website, and analyzed from open sources. Ethics committee approval was not required for this study.

### 2.5. Statistical analysis

We used SPSS Windows 24.0 (SPSS Inc., Chicago, IL) statistical package program for statistical analysis of the data. We determined the distribution patterns of the data with continuous values using the Kolmogorov Smirnov and Shapiro–Wilk tests. We showed the data with continuous values as median and minimum-maximum. In the analysis of data with continuous values, we used the Kruskal Wallis test, Mann–Whitney U test and Wilcoxon test. We showed frequency data as number (n) and percentage (%). We used Pearson Chi-Square and Fisher exact test to analyze frequency data. To determine the consistency of the calculators, “intraclass correlation coefficient (ICC)” analysis was performed for each formula. We determined a *P* value of less than.05 as a significant difference.

## 3. Results

Readability scores for ChatGPT, Bard, and perplexity responses about “Subdural Hematoma” were determined using Calculator 1 and Calculator 2. Text readability scores were statistically compared to the 6th grade reading level and analyzed. The results are presented in Table 2, Table 3, and Table 4, respectively.

**Table 2:** Readability indices for ChatGPT, Bard, and perplexity responses on subdural hematoma and statistical comparison of text content to 6th grade reading level median (minimum—maximum) by using Calculator 1 (https://readabilityformulas.com/free-readability-formula-tests.php).

Readability Indexes	ChatGPT	C6thGRL(p)*^,^†	Bard	C6thGRL(p)*^,^†	Perplexity	C6thGRL(p)*^,^†	p (ChatGPT Bard)‡	p (ChatGPT perplexity)‡	p (perplexity Bard)‡
FRES	19.50 (-3.00–52.00)	**<.001**	42.00 (11.00–65.00)	**<.001**	27.00 (-9.00–55.00)	**<.001**	**<.001**	**<.001**	**<.001**
GFOG	20.45 (14.50–27.00)	**<.001**	15.65 (10.10–21.90)	**<.001**	19.10 (13.60–26.10)	**<.001**	**<.001**	**<.001**	**<.001**
FKGL	16.07 (10.09–19.64)	**<.001**	11.71 (6.86–17.28)	**<.001**	14.73 (9.43–20.02)	**<.001**	**<.001**	**<.001**	**<.001**
SMOG	14.65 (10.56–18.31)	**<.001**	11.14 (7.44–15.56)	**<.001**	13.49 (8.77–18.67)	**<.001**	**<.001**	**<.001**	**<.001**
CL	16.36 (11.22–21.02)	**<.001**	12.04 (8.29–17.43)	**<.001**	15.27 (10.45–23.71)	**<.001**	**<.001**	**<.001**	**<.001**
ARI	17.29 (10.05–21.28)	**<.001**	11.86 (6.50–17.79)	**<.001**	15.55 (8.96–22.84)	**<.001**	**<.001**	**<.001**	**<.001**
LW	17.74 (8.51–22.71)	**<.001**	12.63 (3.40–19.41)	**<.001**	15.38 (5.00–24.43)	**<.001**	**<.001**	**<.001**	**<.001**
FRF	12.77 (10.83–13.99)	**<.001**	11.23 (9.45–12.79)	**<.001**	12.13 (10.27–13.89)	**<.001**	**<.001**	**<.001**	**<.001**
Grade Level	17.00 (11.00–21.00)	**<.001**	12.00 (8.00–17.00)	**<.001**	15.00 (10.00–20.00)	**<.001**	**<.001**	**<.001**	**<.001**
*Reading level*									
Standard/Average n (%)	0 (0%)		2 (2%)		0 (0%)		**<.001**	**<.001**	**<.001**
Fairly difficult to read n (%)	2 (2%)		30 (30%)		2 (2%)				
Difficult to read n (%)	0 (0%)		24 (24%)		14 (14%)				
Very Difficult to read n (%)	93 (93%)		28 (28%)		68 (68%)				
Impossible to Comprehend n (%)	5 (5%)		16 (16%)		16 (16%)				
*Readers age*									
13–15 Years old (Eighth and Ninth Graders) n (%)	0 (0%)		2 (2%)		0 (0%)		**<.001**	**<.001**	**<.001**
14–15 Years old (Ninth to Tenth Graders) n (%)	0 (0%)		5 (5%)		0 (0%)				
15–17 Years old (Tenth to Eleventh Graders) n (%)	0 (0%)		24 (24%)		2 (2%)				
17–18 Years old (Twelfth Graders) n (%)	2 (2%)		27 (27%)		14 (14%)				
18–19 Years old (College Level Entry) n (%)	3 (3%)		16 (16%)		8 (8%)				
21–22 Years Old (college level)	5 (5%)		16 (16%)		15 (15%)				
College Graduate n (%)	90 (90%)		10 (10%)		61 (61%)				

Bold values statistically significant.

ARI = automated readability index, CL = Coleman-Liau score, FKGL = Flesch-Kincaid grade level, FRES = Flesch reading ease score, FRF = Forcast readability formula, GFOG = gunning FOG, LW = Linsear write, SMOG = Simple Measure of Gobbledygook.

* C6thGRL**(p):** Comparison of the responses according to 6th grade reading level **(p**).

† Wilcoxon test.

‡ Chi-Square test for categorical variables and Mann-Whitney U test for continuous variables.

**Table 3:** Readability indices for ChatGPT, Bard, and perplexity responses on subdural hematoma and statistical comparison of text content to 6th grade reading level median (minimum—maximum) by using calculator 2 (https://www.online-utility.org/english/readability_test_and_improve.jsp).

Readability indexes	ChatGPT	C6thGRL(p)*^,^†	Bard	C6thGRL(p)*^,^†	Perplexity	C6thGRL(p)*^,^†	p (ChatGPT Bard)‡	p (ChatGPT perplexity)‡	p (perplexity Bard)‡
FRES	22.39 (1.09–48.66)	**<.001**	36.60 (10.22–59.45)	**<.001**	26.83 (−11.15–53.65)	**<.001**	**<.001**	**.012**	**<.001**
GFOG	16.90 (12.10–23.08)	**<.001**	16.15 (12.07–23.51)	**<.001**	18.91 (12.13–30.56)	**<.001**	**<.001**	**<.001**	**<.001**
FKGL	14.14 (10.16–17.60)	**<.001**	13.13 (9.46–19.75)	**<.001**	15.25 (9.95–27.04)	**<.001**	**<.001**	**.001**	**<.001**
SMOG	15.12 (13.01–18.68)	**<.001**	14.58 (11.22–19.66)	**<.001**	16.53 (12.06–23.12)	**<.001**	**<.001**	**<.001**	**<.001**
CL	15.88 (10.96–20.45)	**<.001**	12.02 (8.06–16.96)	**<.001**	12.61 (7.07–21.66)	**<.001**	**<.001**	**<.001**	.089
ARI	13.58 (9.00–17.06)	**<.001**	11.92 (8.02–19.32)	**<.001**	13.89 (2.55–28.60)	**<.001**	**<.001**	.455	**<.001**

Bold values statistically significant.

ARI = automated readability index, CL = Coleman-Liau score, FKGL = Flesch-Kincaid grade level, FRES = Flesch reading ease score, GFOG = Gunning FOG, LW = Linsear Write, SMOG = Simple Measure of Gobbledygook.

* C6thGRL**(p):** Comparison of the responses according to 6th grade reading level **(p).**

† Wilcoxon test.

‡ Chi-Square test for categorical variables and Mann-Whitney U test for continuous variables.

**Table 4:** Readability indices for ChatGPT, Bard, and perplexity responses on subdural hematoma and statistical comparison of text content to 6th grade reading level median (minimum—maximum) by using the average results obtained from Calculator 1 and Calculator 2.

Readability Indexes	ChatGPT	C6thGRL(p)†,‡	Bard	C6thGRL(p)†,‡	Perplexity	C6thGRL(p)†,‡	p (ChatGPT Bard)§	p (ChatGPT perplexity§	p (perplexity Bard)§
FRES*	21.00 (-1.50–50.33)	**<.001**	39.43 (10.61–61.23)	**<.001**	26.80 (-10.08–54.33)	**<.001**	**<.001**	**.002**	**<.001**
GFOG*	18.71 (13.30–25.04)	**<.001**	15.78 (11.09–21.35)	**<.001**	19.02 (13.32–26.82)	**<.001**	**<.001**	.301	**<.001**
FKGL*	15.28 (10.16–18.62)	**<.001**	12.28 (9.05–17.36)	**<.001**	15.29 (10.62–20.66)	**<.001**	**<.001**	.981	**<.001**
SMOG*	14.85 (11.94–18.50)	**<.001**	12.69 (9.33–16.64)	**<.001**	14.92 (11.28–20.90)	**<.001**	**<.001**	.711	**<.001**
CL*	16.10 (11.09–20.74)	**<.001**	12.03 (8.22–17.20)	**<.001**	13.97 (9.08–22.69)	**<.001**	**<.001**	**<.001**	**<.001**
ARI*	15.38 (9.84–18.80)	**<.001**	11.56 (6.16–17.44)	**<.001**	15.10 (7.41–22.68)	**<.001**	**<.001**	**.048**	**<.001**

Calculator 1: https://readabilityformulas.com/free-readability-formula-tests.php.

Calculator 2: https://www.online-utility.org/english/readability_test_and_improve.jsp.

Bold values statistically significant.

ARI = automated readability index, CL = Coleman-Liau score, FKGL = Flesch-Kincaid grade level, FRES = Flesch reading ease score, GFOG = Gunning FOG, LW = Linsear Write, SMOG = Simple Measure of Gobbledygook.

* [(Calculator 1) + (Calculator 2)]/2.

† C6thGRL**(p):** Comparison of the responses according to 6th grade reading level **(p**).

‡ Wilcoxon test.

§ Chi-Square test for categorical variables and Mann-Whitney U test for continuous variables.

### 3.1. ChatGPT analysis results using the average results obtained from Calculator 1 and Calculator 2

In the text readability analysis of 100 ChatGPT answers, the median FRES was 21.00 (-1.50–50.33), and the median GFOG was 18.71 (13.30–25.04). In addition, the median FKGL and SMOG were 15.28 (10.16–18.62) and 14.85 (11.94–18.50) years of education, respectively. The CL index was 16.10 (11.09–20.74) years of education, the ARI was 15.38 (9.84–18.80) years of education (Table 4).

### 3.2. Bard analysis results using the average results obtained from Calculator 1 and Calculator 2

In the text readability analysis of 100 Bard answers, the median FRES was 39.43 (10.61–61.23), and the median GFOG was 15.78 (11.09–21.35). Also, the median FKGL and SMOG were 12.28 (9.05–17.36) and 12.69 (9.33–16.64) years of education, respectively. The CL index was 12.03 (8.22–17.20) years of education, the ARI was 11.56 (6.16–17.44) years of education (Table 4).

### 3.3. Perplexity analysis results using the average results obtained from Calculator 1 and Calculator 2

In the text readability analysis of 100 perplexity answers, the median FRES was 26.80 (-10.08–54.33), and the median GFOG was 19.02 (13.32–26.82). Also, the median FKGL and SMOG were 15.29 (10.62–20.66) and 14.92 (11.28–20.90) years of education, respectively. The CL index was 13.97 (9.08–22.69) years of education, the ARI was 15.10 (7.41–22.68) years of education (Table 4).

### 3.4. Comparison of ChatGPT, Bard, and perplexity answers to the recommended sixth grade reading level

When the median readability scores of all answers of all artificial intelligent (ChatGPT, Bard, and perplexity) were compared with the sixth-grade reading level, a statistically significant difference was observed in all scores compared to the sixth-grade level (*P* < .001). Relative to all scores, their answers had a readability level above sixth grade. The same statistically significant results were obtained when the comparison was made with Calculator 1, Calculator 2, and the average of both Calculators (Tables 2, 3, and 4).

### 3.5. Comparison of ChatGPT, Bard, and perplexity using the average results obtained from Calculator 1 and Calculator 2

When the readability of the responses of ChatGPT, Bard, and perplexity was compared, it was determined that there was a significant difference between the readability of the responses of tools. Bard responses were more readable than ChatGPT and perplexity responses for all scores evaluated (*P* < .001) (Table 4). Perplexity’s responses were more readable than ChatGPT responses according to FRES, CL and ARI scores (*P* < .001) (Table 4).

### 3.6. Reliability and quality of answers

Bard answers were determined to have the best GQS scores (*P* < .001). Perplexity responses had the best JAMA and modified DISCERN scores (*P* < .001) (Table 5).

**Table 5:** Comparison of modified DISCERN, JAMA and Global Quality Scale (GQS) scores of ChatGPT, Bard and perplexity answers.

	ChatGPT	Bard	Perplexity	p (ChatGPT Bard)*	p (ChatGPT perplexity)*	p (perplexity Bard)*
*Modified DISCERN score*						
0-point, n (%)	5 (5%)	10 (10%)	2 (2%)	.267	**<.001**	**<.001**
1-point, n (%)	6 (6%)	9 (9%)	10 (10%)			
2-point, n (%)	89 (89%)	81 (81%)	8 (8%)			
3-point, n (%)	0 (0%)	0 (0%)	80 (80%)			
*JAMA score*						
0-point, n (%)	0 (0%)	5 (5%)	4 (4%)	.151	**<.001**	**<.001**
1-point, n (%)	95 (95%)	89 (89%)	15 (15%)			
2-point, n (%)	4 (4%)	5 (5%)	81 (81%)			
3-point, n (%)	1 (1%)	1 (1%)	0 (0%)			
*GQS score*						
2-point, n (%)	0 (0%)	7 (7%)	9 (9%)	**<.001**	**<.001**	**<.001**
3-point, n (%)	14 (14%)	11 (11%)	32 (32%)			
4-point, n (%)	66 (66%)	42 (42%)	51 (51%)			
5-point, n (%)	20 (20%)	40 (40%)	8 (8%)			

Bold values statistically significant.

* Chi-Square test for categorical variables.

### 3.7. Intraclass correlation coefficients

FRES, GFOG, FKGL, CL, SMOG, and ARI were calculated using 2 different calculators (https://readabilityformulas.com/free-readability-formula-tests.php, https://www.online-utility.org/english/readability_test_and_improve.jsp).

### 3.8. ICCE for Chat GPT

The intraclass correlation coefficients were 0.913 for FRES, 0.833 for GFOG, 0.742 for KFGL, 0.990 for CL, 0.702 for SMOG, and 0.668 for ARI.

### 3.9. ICCE for Bard

The intraclass correlation coefficients were 0.925 for FRES, 0.794 for GFOG, 0.711 for KFGL, 0.980 for CL, 0.726 for SMOG, and 0.693 for ARI.

### 3.10. ICCE for perplexity

The intraclass correlation coefficients were 0.936 for FRES, 0.721 for GFOG, 0.643 for KFGL, 0.969 for CL, 0.683 for SMOG, and 0.665 for ARI.

## 4. Discussion

We conducted this study to evaluate the readability of the responses provided by ChatGPT, Bard, and perplexity to patients’ questions related to Subdural Hematoma. Our research indicates that the answers provided by ChatGPT, Bard, and perplexity exceed the recommended 6th-grade reading level for articles. Our study is the first and only study to evaluate the readability of patient education materials created by Artificial Intelligence chatbots in the field of Subdural Hematoma.

Readability is an important factor in understanding patient education materials. Readability can be defined as “the ease with which a reader understands a written text.” Complex sentences that contain long words and lengthy phrases can undermine readers’ confidence in what they are learning about medical conditions. Reading materials should be appropriate to the reader’s education level. It is recommended to use sentences of 8 to 10 words to increase the readability of health information. It is reported that simpler words should be used instead of complicated medical terms.^[23–25,28–31]^ When patient education materials on different subjects were examined in previous studies, it was found that their readability levels were above the recommendations, similar to our study.^[23–25,28–31]^

There are no studies in the literature that specifically evaluate online patient education materials related to Subdural Hematoma. However, there is a study evaluating the readability level of emergency radiology websites in which 23 terms including “subdural hematoma” were searched using Google and the first 10 results were recorded and evaluated in terms of readability level.^[32]^ In this study, it was emphasized that the majority of websites containing patient education materials related to emergency radiology, including subdural hematoma, did not meet the recommended reading levels of the NIH and American Medical Association and that the average reader would not be able to fully benefit from these information sources.^[32]^ The study particularly emphasized the link between health literacy and poor health outcomes.^[32]^

Investigating the appropriateness of ChatGPT, Bard, and perplexity answers to medical questions is important because they are used by many patients in a short time to obtain information about their diagnosed diseases.^[33]^ To our knowledge, no studies have evaluated the readability of ChatGPT, Bard, and perplexity subdural hematoma responses.

The readability and quality assessment of ChatGPT or Bard in healthcare has been studied in several studies with conflicting results. In one study, conducted in the field of radiology, McCarthy et al^[34]^ examined the performance of ChatGPT in providing educational material to patients on interventional radiology and compared ChatGPT output to that provided by a Societal website. The researchers highlighted that the content on both ChatGPT and the Societal website was significantly above the recommended level for patient education. They stated that the outputs of the ChatGPT platform were longer and more difficult to read. In this study, it was determined that ChatGPT’s answers are difficult and complex to read and that it is necessary to be a university graduate in order to read them. Researchers also specifically stated that 11.5% of the 104 questions asked to ChatGPT were answered incorrectly.^[34]^ Golan et al^[35]^ assessed the quality of online content on shock wave therapy for erectile dysfunction, and found that ChatGPT’s performance in assessing the quality of text content was inadequate and was not consistent with standards set by human evaluators and reliable tools.^[35]^ Momenaei et al^[36]^ evaluated the relevance and readability of the medical information provided by ChatGPT-4 regarding common vitreoretinal surgeries for retinal detachments (RDs), macular holes (MHs), and epiretinal membranes (ERMs). They noted that the mean Flesch Kincaid Grade Level and Flesch Reading Ease Score were 14.1 ± 2.6 and 32.3 ± 10.8 for RD, 14 ± 1.3 and 34.4 ± 7 for MD, 14.8 ± 1.3 and 28.1 ± 7.5 for ERM.^[36]^ The results of this study showed that the answers were difficult or very difficult to read for the average lay person and that a college degree would be required to understand the material.^[36]^ Johnson et al^[37]^ compared the answers of ChatGPT and “National Cancer Institute” by using the questions on the “Common Cancer Myths and Misconceptions” web page, and found that ChatGPT provided accurate information about common cancer myths and misconceptions.^[37]^ According to a study conducted by Musheyev et al^[38]^ the responses provided by ChatGPT, perplexity, Chat Sonic, and Microsoft Bing AI on the topic of urological cancers were found to be fairly difficult to read, moderately difficult to understand, and lacked clear instructions for users on how to take action.^[38]^ In another study where questions about bladder cancer, prostate cancer, kidney cancer, benign prostatic hypertrophy and urinary stones were questioned via ChatGPT, it was determined that all treatment responses had a medium quality score in DISCERN. The researchers specifically stated that ChatGPT information should be used carefully because the chatbot does not disclose the sources of information and may contain bias even in simple questions regarding the basics of urological diseases.^[39]^ In another study evaluating ChatGPT responses for benign paroxysmal positional vertigo, it was emphasized that ChatGPT responses were harder to read, lower quality, and harder to understand than the information in Google searches.^[40]^ In a study planning to analyze the quality and readability of available information on shoulder stabilization surgery provided by ChatGPT, it was reported that the answers given were generally of good quality. However, the researchers particularly emphasized that the reading level must be high in order to understand the information provided by ChatGPT. It was also reported that no source material was cited and it was unclear where the answers came from.^[41]^ In another study, patient questions were developed based on Google Trends regarding oncological, benign and emergency conditions in the field of urology and these questions were asked to ChatGPT. ChatGPT responses were evaluated for accuracy, comprehensiveness, clarity and readability. In this study, it was reported that 77.8% of ChatGPT responses were of appropriate quality, the Flesch Reading Ease score of the responses was determined as 35.5 ± 10.2, and the average Flesh-Kincaid Reading Class Level score was determined as 13.5 ± 1.74. Researchers have particularly emphasized that natural language processors have limitations as a source of medical information.^[42]^

All of the answers given by all 3 chat robots we evaluated in our study regarding Subdural Hematoma were found to be much higher than the recommended sixth grade level. Google Bard responses were found to be more readable for all formulas evaluated. It was determined that a university degree was required to understand Chat GPT and perplexity responses. The data of our study are also compatible with previous literature.

The artificial intelligence responses investigated in our study have DISCERN and JAMA scores indicating low quality. When the reasons for this were examined, it was seen that there were deficiencies in artificial intelligence responses in terms of DISCERN and JAMA evaluation criteria. Not stating conflicts of interest, not stating the last update date, and not stating the bibliography of the artificial intelligence responses evaluated in our study are among the reasons for low scores in DISCERN and JAMA evaluations. All these factors resulted in low scores for both DISCERN and JAMA. Low-medium quality DISCERN and JAMA scores and difficult readability have been reported in studies on different subjects, similar to our study.^[35,38–41]^ However, in our study, among the artificial intelligence chat robots, perplexity especially attracts attention with its high DISCERN and JAMA scores. The fact that the answers of this artificial intelligence chatbot contain references has caused its DISCERN and JAMA scores to be high. In order for artificial intelligence chatbots to provide better quality information, they must provide answers that comply with DISCERN and JAMA criteria.

## 5. Limitations

This study has some notable limitations. First, we used ChatGPT, Bard, and perplexity as chatbots. The reason we selected these chatbots was their popularity, availability, and free usage. Second, this study only included answers from January 3, 2024. Changes in the answers after asking the questions again on the following days were not included in this study. In this study, we only recorded the sufficiency of the answers. The accuracy of the answers was not evaluated in our study. Comprehensibility of the answers (PEMAT score) was also not included in our study. Future studies can evaluate the accuracy of the answers and PEMAT scores. Further studies are needed for other considerations such as ethics, privacy, bias, and discrimination.

The questions determined in our study were asked directly to the artificial intelligence chat robot, as in previous studies. No additional directives have been given.^[35–40]^ In these studies, it is particularly emphasized that the readability values of the information provided by artificial intelligence are above the recommended sixth grade level.^[35–40]^ As a limitation, the artificial intelligence chat robot is not asked to organize its answers in accordance with a certain grade level. However, a fairly recent study evaluated “original” and “simplified” responses.^[43]^ In this study,^[43]^ ChatGPT was asked to “simplify” answers to 25 questions about breast cancer down to a sixth-grade reading level. The researchers evaluated the “simplified responses” for clinical relevance. All “original” and “simplified responses” included in the study were also evaluated for readability. The researchers reported that while all of the “simplified responses” met criteria for adequate ease of reading, only 8% of the “original response” met readability criteria. However, researchers have emphasized that “simplified responses” can increase the potential for generating misinformation.^[43]^ This issue should also be taken into consideration in future studies.

However, the study has some strengths. In this study, 2 calculators were used to evaluate the readability of websites. To our knowledge, there is no gold standard calculator for readability. We used 2 publicly available and widely used online calculators. We also assessed whether the 2 calculators were compatible with each other.

## 6. Conclusion

ChatGPT, Bard, and perplexity may offer the possibility of improving health outcomes and patient satisfaction in Subdural Hematoma by serving as an interactive tool for providing medical information online. However, the current capabilities of ChatGPT, Bard, and perplexity are not sufficient for the readability of responses about Subdural Hematoma. Efforts should be made to ensure that responses to all 3 are at an appropriate level of readability.

## Author contributions

**Conceptualization:** Şanser Gül.

**Data curation:** İsmail Erdemir, Volkan Hanci.

**Formal analysis:** Volkan Hanci.

**Funding acquisition:** Şanser Gül.

**Investigation:** Evren Aydoğmuş, Yavuz Selim Erkoç.

**Methodology:** Şanser Gül, Volkan Hanci.

**Resources:** Yavuz Selim Erkoç.

**Supervision:** Şanser Gül.

**Software:** Evren Aydoğmuş.

**Writing – original draft:** İsmail Erdemir, Volkan Hanci, Evren Aydoğmuş.
